# Calcium dobesilate reduces endothelin-1 and high-sensitivity C-reactive protein serum levels in patients with diabetic retinopathy

**Published:** 2013-01-10

**Authors:** Alireza Javadzadeh, Amir Ghorbanihaghjo, Farzad Hami Adl, Dima Andalib, Hassan Khojasteh-Jafari, Kamyar Ghabili

**Affiliations:** 1 Nikookari Eye Hospital, Tabriz University of Medical Sciences, Tabriz, Iran; 2 Drug Applied Res earch Center, Tabriz University of Medical Sciences, Tabriz, Iran; 3 Farabi Eye Hospital, Tehran University of Medical Sciences, Tehran, Iran; 4 Physical Medicine and Rehabilitation Research Center, Tabriz University of Medical Sciences, Tabriz, Iran; 5 Young Researchers Club, Tabriz Branch, Islamic Azad University, Tabriz, Iran

## Abstract

**Purpose:**

To determine the benefits of calcium dobesilate (CaD) administration on endothelial function and inflammatory status in patients with diabetic retinopathy through measurement of serum levels of endothelin-1 and high-sensitivity C-reactive protein (hsCRP).

**Methods:**

In a double-blind, randomized clinical trial, 90 patients with either severe nonproliferative or proliferative diabetic retinopathy and with blood glucose level of 120–200 mg/dl were randomly allocated to treatment with either CaD tablets (500 mg daily) or placebo for 3 months. Visual acuity, intraocular pressure, and macular status were performed before the study. The serum levels of endothelin-1 and hsCRP were evaluated in both groups before and at the third month of the trial.

**Results:**

The median serum level of hsCRP significantly differed between the groups 3 months following the CaD or placebo administration (2.2 mg/l in the CaD group versus 3.7 mg/l in the placebo group, p=0.01). The mean endothelin-1 serum level was 0.69±0.32 pg/ml in the CaD group and 0.86±0.30 pg/ml in the placebo group (p=0.01). Furthermore, in the CaD group, the serum levels of both endothelin-1 and hsCRP were significantly decreased 3 months after administration of CaD (p<0.001).

**Conclusions:**

Administration of the CaD in the patients with diabetic retinopathy may reduce the serum levels of endothelin-1 and hsCRP. This might imply amelioration of the endothelial function and inflammatory status following CaD therapy in these patients.

## Introduction

Diabetes mellitus is one of the most frequent chronic diseases, with an increase in the global prevalence [1-3]. Among the microvascular complications of the diabetes mellitus, diabetic retinopathy (DR) might lead to acquired blindness in adults [4,5]. Hyperglycemia, as a major risk factor in development of DR, chiefly targets vascular endothelial cells with unknown underlying mechanisms [6,7]. Moreover, defects in autoregulation of retinal blood flow play a key role in the formation of DR. Pericytes of retinal vessels are the most important regulators of vascular tone in retinal capillaries. These cells include receptors of contractor proteins, among which endothelin-1 is of the utmost importance. In addition to its potent vasoconstricting feature, endothelin-1 acts as a powerful mitogen of smooth muscles [8]. Furthermore, several animal and human investigations have revealed endothelin-1’s role in the pathogenesis of DR [9-12]. Accordingly, the activation of the endothelin-1 system in DR has been highlighted [13,14]. On the other hand, oxidative and inflammatory mediators such as C-reactive protein (CRP) have been deemed in the pathogenesis of DR [15-18]. CRP is known to decrease nitric oxide, to increase endothelin-1 in the endothelial cells, reactive oxygen species in monocytes, inducible nitric oxide production, and to upregulate angiotensin II type 1 receptor in the vascular smooth muscle cells. The latter results in increased reactive oxygen species and proliferation of the vascular smooth muscle cell [15,19].

Owing to the role of oxidative mediators in the pathogenesis of DR as well as complications of current treatments such as laser photocoagulation, antioxidants are applied in the treatment of DR [20-22]. Among these, calcium dobesilate (CaD; calcium 2,5-dihydroxybenzenesulfonate or doxium) has been widely prescribed to treat chronic venous insufficiency, hemorrhoids and to prevent the progression of DR [23,24]. Animal studies have revealed that CaD stimulates the synthesis of endothelium-derived vasoactive mediators such as nitric oxide. These substances, apart from their antioxidative roles, are capable of improving the endothelial function [25-27]. In human studies, the role of CaD in reducing blood viscosity and microvascular permeability has been highlighted [28]. Nonetheless, the advantage of CaD in the treatment of diabetic retinopathy is still a matter of debate in human studies.

To the best of our knowledge, there is no investigation demonstrating the effect of CaD on the serum levels of both endothelin-1 and high-sensitivity CRP (hsCRP) in patients with DR, in the literature available in English. Therefore, the aim of the present study was to determine the benefits of CaD administration on the endothelial function and inflammatory status in these patients through the measurement of the serum levels of endothelin-1 and hsCRP, respectively.

## Methods

In a double-blind randomized clinical trial lasting 17 months, from May 2008 to September 2009, 124 patients with either severe nonproliferative DR or proliferative DR (40–70 years) and with blood glucose levels of 120–200 mg/dl were eligible for inclusion. Thirty-four patients were excluded from the study, and the remaining patients were randomly allocated, to treatment with either CaD or placebo, by computer software (Graphpad; GraphPad Software Inc., San Diego,

CA). Exclusion criteria were history of doxium administration, allergy to doxium, active liver disease, and/or unexplained elevation of liver enzymes. Moreover, if any therapeutic intervention such as photocoagulation were to be required for a patient during the study, the patient would be excluded from the trial to receive the treatment. To reduce the effect of nutrition and medication, all patients were put on the same drug and nutritional regimens. The Institutional Review Board approved the project and investigators followed the principles of the Declaration of Helskinki. Informed consent was obtained from each patient.

Complete ocular examination including Snellen chart visual acuity measurement, intraocular pressure evaluation by applanation tonometry, and examination with slit lamp. Macular status was evaluated using a slit lamp with a super-field indirect lens (Haag-Streit 900®; Haag-Streit AG, Koeniz, Switzerland), fundus photography, and fluorescein angiography (Imagenet 2000; Topcon Corp., Tokyo, Japan). The CaD group received doxium tablets (500 mg daily) for 3 months. The placebo group was put on identical placebo tablets daily for 3 months. Serum levels of endothelin-1 and hsCRP were evaluated in both CaD and placebo groups before the trial and at third month of CaD or placebo administration. Blood samples were collected into EDTA tubes after an overnight fasting, homogenized, and the liquid centrifuged (3,000 rpm) for 10 min at 4 °C. The supernatant was frozen in −80 °C, lyophilized for 48 h, and stored for further laboratory measurements. High-sensitivity CRP was measured by nephelometry, a latex particle-enhanced immunoassay (Pars Azmoon, Tehran, Iran). Measurement of the serum endothelin-1 was performed by human endothelin-1 immunoassay kit (R&D systems, Minneapolis, MN). Serum levels of fasting blood glucose, total cholesterol, high-density lipoprotein cholesterol, triglyceride, aspartate aminotransferase (AST), alanine aminotransferase, urea, and creatinine were determined using commercial reagents with an automated chemical analyzer (Abbott analyzer, Abbott Laboratories, Abbott Park, Chicago, IL). Low-density lipoprotein cholesterol was calculated using the Friedewald equation [29].

Data were presented as mean±standard deviation (SD) or as median (interquartile range). Statistical analysis was performed with the Statistical Package for Social Sciences for Windows (Version 16, SPSS Inc., Chicago, IL) using the chi-square test, Fisher’s exact test, Mann–Whitney U test, Wilcoxon signed-rank test, independent-samples *t* test, and paired-samples *t* test, as appropriate. A p value of <0.05 was considered statistically significant.

## Results

Ninety of 124 patients with DR were evaluated: 45 patients in group CaD and 45 cases in the placebo group. The demographic data and baseline characteristics of these patients are shown in Table 1. There were no differences in gender, age, type of DR, body mass index (BMI), systolic and diastolic blood pressure, duration of diabetes mellitus (DM) and DR, or visual acuity (Table 1, p>0.05). Prior to the CaD or placebo administration, patients in both groups did not differ in fasting blood glucose, lipid profile, aminotransferases, urea, creatinine, endothelin-1, or hsCRP (Table 2, p>0.05). Three months after the CaD or placebo administration, the serum levels of total cholesterol (p=0.02, independent-samples *t* test) and triglyceride (p=0.01, Mann–Whitney U test) showed significant reduction in patients receiving CaD (Table 2). The median serum level of hsCRP significantly differed between groups 3 months following the CaD or placebo administration (2.2 mg/l [interquartile range 0.85–4.15] in the CaD group versus 3.7 mg/l [interquartile range 1.95–5.95] in the placebo group; p=0.01, Mann–Whitney U test, Table 2). The mean endothelin-1 serum level was 0.69±0.32 pg/ml in the CaD group and 0.86±0.30 in the placebo group (p=0.01, independent-samples *t* test, Table 2). Furthermore, in the CaD group, the serum levels of both endothelin-1 and hsCRP were significantly decreased 3 months after administration of CaD (p<0.001, Table 2).

**Table 1:** Demographic and baseline characteristics of the patients [mean±SD (median, interquartile range)].

Parameters	Group Ca-D (n=45)	Group placebo (n=45)	P value
Gender (male:female)	0.604861	0.686806	0.82
Age (years)	59.04±8.29 (60, 53–64)	59.33±5.83 (60, 55–64)	0.84
Proliferative DR: n (%)	37 (82.2)	36 (80)	1
Severe non-proliferative DR: n (%)	8 (17.8)	9 (20)	1
BMI (kg/m^2^)	27.56±4.27 (27.74,24.18–30.45)	27.51±4.44(27.63,24.26–29.82)	0.95
Systolic BP (mmHg)	137.55±21.60 (140,120–150)	137.00±18.41(135, 125–142.5)	0.89
Diastolic BP (mmHg)	77.55±12.21 (80, 67.5–80)	76.88±10.72 (80, 70–82.5)	0.58
Duration of DR (years)	5.13±2.88 (5, 2.5–8)	4.74±3.06 (4, 3–6)	0.53
Duration of DM (years)	16.28±6.93 (15, 10–20)	15.48±5.99 (15, 11–20)	0.56
Visual acuity (logMAR)	0.69±0.25 (0.62, 0.45–0.95)	0.72±0.23 (0.75, 0.53–1)	0.42

Ca-D, calcium dobesilate; DR, diabetic retinopathy; BMI, body mass index; BP, blood pressure; DM, diabetes mellitus; logMAR, logarithm of the minimum angle of resolution.

**Table 2:** Laboratory parameters before and after Ca-D or placebo administration [mean±SD (median, interquartile range)].

Laboratory parameters†	Group Ca-D	Group Placebo	P value	Group Ca-D	Group placebo	P value before	P value‡ after
FBS	133.7±57.1(130,89.5–160.5)	133±66.2 (105, 76.5–194.5)	0.95	138.9±47.3 (144, 96–167.5)	142.1±64.3(125,91.5–185)	0.79	0.5
Total cholesterol	188.4±51.4(185,152–221.5)	202.9±58.5 (192, 160–242.5)	0.21	183.6±38.7(188,157.5–214)	205.2±52.2(205,160–242.5)	0.02*	0.45
HDL-C	44±6.4 (44, 38–48)	44.2±6.6 (44, 38–48)	0.89	44.3±5.8 (44, 40–48)	42.3±5.9(42,38.5–45)	0.1	0.78
LDL-C	113.8±39.2(115,77–149)	125.2±54.3 (111, 90.5–170)	0.25	109±36.6 (113,77–130.5)	125.6±47(115,89.5–148.5)	0.06	0.32
Triglyceride	169.1±72.7(152, 119.5–213)	193.4±110 (177, 116–220)	0.46	161.2±61.6(160, 112–196)	207.4±106.3(194, 135–275.5)	0.01*	0.29
AST	31.2±10.4(31,23–39.5)	33.2±9.8 (33, 25.5–41)	0.34	29.6±8.3(30,22.5–36.5)	33.5±7.9 (34, 28–39)	0.02*	0.08
ALT	34.5±9.7 (32, 27–42)	35.2±10.5 (35, 28–41)	0.73	33.3±9 (32, 26.5–40)	36.2±7.8(38,28–43.5)	0.11	0.1
Urea	38.4±16.7(35,27–46.5)	35.3±13 (34, 29.5–38)	0.44	39.1±15.7 (35,30.5–45)	40.8±19.7 (36,36–45)	0.79	0.91
Creatinine	1.2±0.5 (1.1, 0.8–1.4)	1.7±4.4 (1.1, 0.9–1.2)	0.59	1.4±1.5 (1.1, 1–1.3)	3.1±12.1 (1.2, 1–1.4)	0.61	0.52
Endothelin-1	0.8±0.2(0.83,0.7–1.01)	0.7±0.3 (0.77, 0.48–0.95)	0.1	0.6±0.3 (0.68,0.47–0.90)	0.8±0.3 (0.9,0.64–1.09)	0.01*	<0.001*
hsCRP	5.6±6.9 (3.4, 1.3–7.5)	3.8±3.6 (2.9, 1.3–5.1)	0.44	3.2±3.2 (2.2, 0.85–4.15)	4.6±3.7 (3.7, 1.95–5.95)	0.01*	<0.001*

^†^All the units are mg/dl, except endothelin-1 (pg/ml) and hsCRP (mg/l). ^‡^P value related to the comparisons between the Ca-D group before and after the treatment. Ca-D, calcium dobesilate; FBS, fasting blood glucose; HDL-C, high-density lipoprotein cholesterol; AST, aspartate aminotransferase; ALT, alanine aminotransferase; hsCRP, high sensitivity C-reactive protein. *Statistically significant.

## Discussion

The present study showed that CaD administration to patients with DR might reduce the serum level of endothelin-1 and hsCRP. This may imply amelioration of vascular dysfunction and inhibition of the inflammatory process following CaD therapy in DR patients. The results of this study regarding the endothelin-1 and hsCRP complement those of previous publications. In a study on patients with DR, Zhong and Guo [30] observed reduced plasma levels of endothelin following treatment with doxium. In addition, Gao et al. [31] suggested a protective role of CaD toward the vascular endothelium; they found decreased plasma endothelin levels after CaD administration for streptozotocin-induced diabetic nephropathy in rats. On the other hand, Xia et al. [32] concluded that CaD might reduce the serum levels of hsCRP and improve the microinflammatory state in maintenance hemodialysis patients. Moreover, in a study on early diabetic nephropathy patients receiving CaD for 12 weeks, Dong et al. [33] detected decreased serum levels of endothelin-1 and increased levels of nitric oxide, which in turn led to a delay in the development of diabetic nephropathy. Nevertheless, to the best our knowledge, the present research is the first investigation in the English literature to study the effects of CaD on the serum levels of both endothelin-1 and hsCRP in patients with DR.

Ocular blood flow is autoregulated through non-nervous mechanisms, including endothelin-1 [13]. However, any imbalance in the endothelin-1 and other mediators contributes to retinal hemodynamic abnormality in DR [34,35]. Several investigations in streptozotocin-induced diabetic rats and human studies have revealed a role for endothelin-1 in the pathogenesis of DR [9-12]. Moreover, Shaw et al. [13] detected that endothelin-1 antagonist might prevent the development of DR in a genetic mouse model of non-obese diabetes mellitus. Their findings, along with the similar results by Masuzawa et al. [14], have highlighted the activation of the endothelin-1 system in DR [13]. On the other hand, increased plasma levels of CRP, a key marker of inflammation, have been detected in patients with diabetes mellitus [15,36,37]. Inflammatory and atherogenic effects of CRP may result in increased reactive oxygen species and proliferation of the vascular smooth muscle cell [15,19]. Additionally, recent evidence is suggestive of an association between the serum levels of endothelin-1 and hsCRP and the course and progression of DR [38-40].

There is still controversy over the advantages of CaD in the treatment of DR in human studies. Numerous reports have highlighted the role of CaD in slowing the progression of DR [41-46]. Garay and colleagues [47] concluded that CaD might protect against diabetic endothelial dysfunction, reduce apoptosis, and retard the local proliferation of the vascular cells—in addition to its antioxidant activity. In contrast, Larsen et al. [48] and Rasch [49] failed to produce any evidence in favor of the benefits of CaD on the capillary resistance in diabetic patients or on the progression of DR. Likewise, in a recent double-blind multicenter trial, Haritoglou et al. [50] revealed that CaD could not prevent or reduce the development of macular edema during a 5-year follow-up period in patients with nonproliferative DR. In a trial on obese nondiabetic male smokers, Schram et al. [51] found no ameliorating effects of CaD (1,000 mg/d), either on endothelial function, as determined by endothelium-dependent vasodilation and markers of endothelial function, or on markers of oxidation. They attributed the discrepancy between their findings and those previously found in animal studies to the different dosing of CaD [51]. However, in the present study, we found reduced serum levels of endothelin-1 and hsCRP, indicating amelioration of the vascular dysfunction and inhibition of the inflammatory process following CaD therapy in DR patients.

Apart from the beneficial effect of CaD on endothelin-1 and hsCRP in the present study, we found decreased serum levels of total cholesterol, triglycerides, and AST following the CaD administration (500 mg/d). Our finding about reduced levels of total cholesterol is comparable to the finding of Benarroch et al. [52], in which CaD (1,500 mg/d) was administered to patients with DR for 3 months. In contrast, Beyer et al. [53] reported unchanged levels of both cholesterol and triglycerides in diabetic patients 6 months after treatment with CaD (750 mg/d). We believe that a plausible explanation for the discrepancy between these findings may lie in the dosing and duration of treatment with CaD.

This study has a limitation: we did not measure the clinical parameters (e.g., visual acuity) following the CaD treatment. Therefore, assessment of the possible relationship between various parameters (e.g., the serum endothelin-1 and hsCRP) and visual acuity—among all the patients at baseline and in each group separately after the treatment—could not be performed. On the other hand, the authors believe that evaluating the effects of CaD on the serum levels of both endothelin-1 and hsCRP in patients with DR—effects that imply endothelial function and inflammatory status—should be highlighted as an advantage of the current study.

In conclusion, our data suggest that administration of CaD to patients with DR may reduce the serum levels of endothelin-1, hsCRP, total cholesterol, triglycerides, and AST. This finding not only suggests the beneficial effect of CaD on vascular function and the inflammatory process, but it also points to the lipid-lowering features of CaD. Further investigations are recommended to assess the benefits of CaD on clinical parameters such as the visual acuity and photocoagulation indications in these patients.
